# Strategies Used for the Discovery of New Microbial Metabolites with Antibiotic Activity

**DOI:** 10.3390/molecules30132868

**Published:** 2025-07-06

**Authors:** Pablo Dasí-Delgado, Cecilia Andreu, Marcel·lí del Olmo

**Affiliations:** 1 Departament de Bioquímica i Biologia Molecular, Universitat de València (UVEG), Dr. Moliner 50, E-46100 Burjassot, València, Spain; dasipablo@icloud.com; 2 Departament de Química Orgànica, Universitat de València (UVEG), Vicent Andrés Estellés s.n., E-46100 Burjassot, València, Spain; cecilia.andreu@uv.es

**Keywords:** antibiotic, antimicrobial resistance, artificial intelligence, drug discovery, extremophiles, isolation Chip, metagenomics, microorganisms, natural products, secondary metabolites

## Abstract

The discovery of new microbial metabolites is essential to combat the alarming rise in antimicrobial resistance and to meet emerging medical needs. This work critically reviews current strategies for identifying antimicrobial compounds, emphasizing the potential of microorganisms as a rich source of bioactive secondary metabolites. This review explores innovative methods, such as investigating extreme environments where adverse conditions favor the emergence of unique metabolites; developing techniques, like the iChip, to cultivate previously uncultivable bacteria; using metagenomics to analyze complex samples that are difficult to isolate; and integrates artificial intelligence to accelerate genomic mining, structural prediction, and drug discovery optimization processes. The importance of overcoming current challenges, such as replicating findings, low research investment, and the lack of adapted collection technologies, is also emphasized. Additionally, this work analyzes the crucial role of bacterial resistance and the necessity of a holistic approach involving new technologies, sustained investment, and interdisciplinary collaboration. This work emphasizes not only the current state of metabolite discovery but also the challenges that must be addressed to ensure a continuous flow of new therapeutic molecules in the coming decades.

## 1. Introduction

**Figure 1:**
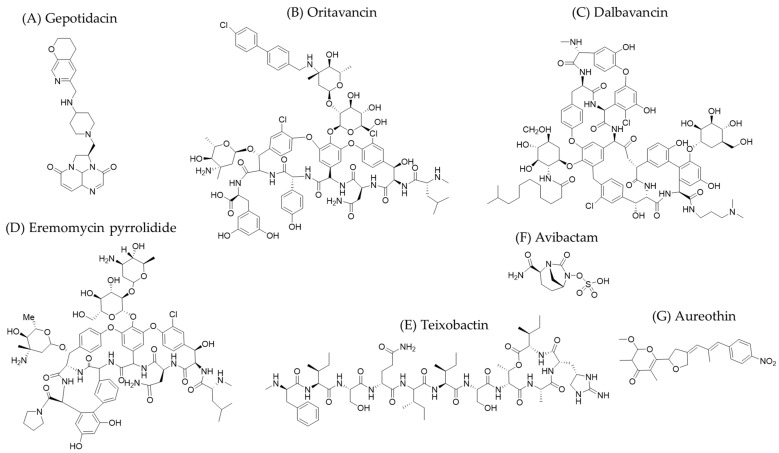
The chemical structures of several recently developed metabolites. (**A**) Gepotidacin is an antibacterial compound approved by FDA in 2025. (**B**) Oritavancin, (**C**) dalbavancin, and (**D**) eremomycin pyrrolidide are glycopeptides used to treat bacterial infections. (**E**) Teixobactin is a cyclodipeptide employed for infections produced by MRSA. (**F**) Avibactam is a penicillin derivative utilized against Gram-negative bacteria. (**G**) Aureothin is a polyketide antibiotic and antiviral agent.

Historically, microorganisms have played a fundamental role in the progress of the human species. They produce both primary (e.g., amino acids) and secondary metabolites (e.g., antibiotics), the latter being of particular pharmacological relevance. The first major milestone in the history of antibiotics undoubtedly took place in 1928, when Alexander Fleming discovered the bactericidal potential of a compound that had formed in an open Petri dish left near a window. Tan & Tatsumura quote his famous statement, “I did not invent penicillin. Nature did that” [1]. This finding revolutionized medicine, initiating what is known as the golden age of antibiotics and laying the foundation for future disease control. Over the following decades, 70% of new antibiotics were isolated from *Streptomyces* species [2]. This genus of bacteria has also yielded the herbicide glyphosate [3] and the antiparasitic avermectin, which is of interest in agriculture [4]. However, the discovery of new compounds with antibacterial activity has decreased in recent decades. Since 2010, only 29 new antibiotics have received marketing authorization, most of which are modifications of existing classes [5]. In 2022, a World Health Organization report indicated that 80 new antimicrobial drugs were in the clinical or preclinical phase: 46 were traditional (direct-acting small molecules), and 34 were non-traditional (based on emerging therapies). Of these, 34 were in Phase 1 trials, 29 in Phase 2, 14 in Phase 3, and 3 in regulatory evaluation ([5] and references therein). One of these agents, Gepotidacin (Figure 1), is a first-in-class, bactericidal, triazaacenaphthylene antibacterial that inhibits bacterial DNA replication. It was approved by the Food and Drug Administration (FDA) in 2025 for treating uncomplicated urinary tract infections. It is a DNA gyrase inhibitor without cross-resistance ([6] and references therein).

There are different types of antibacterials that vary in their mechanisms of action ([7] and references therein). They are classified into two main groups, bactericidal and bacteriostatic. However, this differentiation is becoming increasingly blurred, as the same antibiotic can act in both ways at the same time or depending on the organism. Table 1 shows the different classes of antibiotics according to their chemical structure and target. The most widely used are beta-lactams, which have a lactam ring and inhibit the bacterial surface enzymes involved in peptidoglycan wall structuring. Penicillins are included in this class of antibiotics [8]. Another well-known example is protein synthesis inhibitors, such as tetracyclines, which prevent aminoacyl-tRNA from binding to the ribosome. Compounds that modify or affect the structure of nucleic acids also play a crucial role, with quinolones and rifamycins standing out in this group. The former inhibit the action of topoisomerases II and IV, while the latter affect RNA synthesis by binding to the beta subunit of RNA polymerase ([7] and references therein).

### 1.1. Microbial Secondary Metabolites

Advances in screening, molecular biology, and bioinformatics analyses have led to the discovery of more than one million natural products [2,9]. Over fifty thousand of these products are secondary metabolites, which are low-molecular-mass molecules derived from main biosynthetic pathways that may or may not exhibit bioactivity. These molecules are of high interest to the pharmaceutical industry because of their potential antibacterial, anti-inflammatory, anticancer, and herbicidal properties [10]. Although they seem unnecessary for the producing organisms, their vital importance has been demonstrated in relation to interactions within biofilms or other microbial communities [2,11]. The most notable features of secondary metabolites are their incredible variety of chemical structures, their presence in different types of organisms, and their multiple bioactivities [2]. The term “secondary metabolite” was introduced later, but the first crystalline fungal product considered as such was mycophenolic acid. Bastolomeo Gosio discovered it in 1896 [12].

Certain numerical data highlight the significance of microorganisms and the secondary metabolites they produce. The production of specialized metabolites is known to consume up to 15% of the genome content of certain microorganisms [13]. Currently, it is estimated that 53% of FDA-approved drugs based on natural products come from microorganisms [14]. Those symbiotically associated with sponges alone are responsible for producing up to 3300 bioactive compounds [15]. Additionally, species belonging to the order *Actinomycetales* produce 45% of the total bioactive microbial metabolites, totaling around 10,000. Approximately 7600 of these are from the genus *Streptomyces*, and 2500 are from rare actinobacteria [2,16,17]. Notably, antivirals alone accounted for approximately 20% of the market as early as the 2000s [18]. In 2018, antibacterial drugs such as cephalosporins, which are isolated from the *Acremonium* fungus, as well as their modified derivatives (ceftaroline), approached a USD 140 million market share in the United States [10].

**Table 1:** Main targets, mechanisms of action, and classes of antibiotics ^1^.

Bacterial Target	Mechanism	Antibiotic
Cell wall	Inhibition of its synthesis	Beta-lactams Glycopeptides Bacitracin Isoxazolidinones
DNA synthesis	Inhibition of enzymes that control DNA topology	Quinolones Nitroimidazoles
RNA synthesis	Inhibition of the RNA polymerase	Rifamycins Nitrofurans
Protein synthesis	Translation blocking	Aminoglycosides Tetracyclines Macrolides Lincosamides Fusidic acid Phenicols Streptogramins Oxazolidinones Glycylcyclines Mupirocin
Plasma membrane	Changes in permeability	Polymixins Lipopeptides Ionophores
Folic acid synthesis	Inhibition of enzymes needed for this process	Sulfonamides Diaminopyrimidines

^1^ Data are from [7,19].

It is true that microbial products are not always used strictly as drugs but rather undergo different modifications. For this reason, they can be classified into three groups: (i) the original products, (ii) products derived from them or chemically synthesized, and (iii) synthetic products based on their structure [10]. Ultimately, microbial products derived from secondary metabolites are found in many products, especially antibiotics, antifungals, and antivirals, either directly or indirectly.

### 1.2. Examples of Compounds with Antibiotic Activity Derived from Secondary Metabolites

Figure 1 shows lipoglycopeptides, such as oritavancin and dalbavancin, which are antibiotics approved in 2014 for use against vancomycin-resistant Gram-positive bacteria [10,20]. Dalbavancin binds to the D-alanyl-D-alanine dipeptide terminus of growing bacterial peptidoglycan. This sequesters the substrate from the transpeptidation and transglycosylation reactions of peptidoglycan synthesis. Oritavancin, on the other hand, acts by disrupting the integrity of the bacterial membrane and inhibiting bacterial RNA synthesis. It has a lower MIC (minimal inhibitory concentration) value than vancomycin, making it more effective at lower doses. Another example of a glycopeptide is eremomycin pyrrolidide (Figure 1), which has the same mechanism of action as dalbavancin. It exhibits high activity against *Staphylococcus* and *Enterococcus* without causing pseudoallergic reactions [21]. Teixobactin (Figure 1), an 11-residue cyclodepsipeptide, is an antibiotic isolated from *Elephtheria terrae* that treats infections caused by Gram-positive bacteria, including methicillin-resistant *Staphylococcus aureus* (MRSA). This compound binds to the pyrophosphate group of lipid II and related cell wall precursors. It inhibits cell wall biosynthesis, ultimately killing bacteria by aggregating on the cell surface and lysing them [22].

Avibactam (Figure 1) is also worth mentioning. It is a penicillin derivative and an anti-infective agent that acts against Gram-negative bacteria. It was developed to work with other beta-lactams to treat *Klebsiella pneumoniae* [23]. Avibactam inhibits class A β-lactamases by forming a covalent bond with the serine in their active center. It behaves as a reversible (non-suicidal) inhibitor against most of these enzymes.

Aureothin (Figure 1) is another compound that should be mentioned. It is a nitroaryl polyketide metabolite from *Streptomyces thioluteus* that acts as an antibacterial, antifungal, antineoplastic, and antiparasitic agent. It is used to treat human immunodeficiency virus (HIV) infections [10,24]. Aureothin has a complex structure containing a γ-pyrone ring, a 4-nitrophenyl group, a tetrahydrofuran ring, and a conjugated diene system. The 4-nitrophenyl group contributes to its ability to bind to cellular targets, such as ATP-dependent RNA helicases. This disrupts the synthesis of proteins essential for infection. However, its use is limited due to its high toxicity.

This review focuses on microbial metabolites with antibacterial activity. However, we cannot conclude this section without mentioning natural product-derived compounds used to treat infections caused by other microorganisms. These include the antifungals caspofungin and micafungin [25,26,27,28] and the antivirals cytarabine, vidarabine, and xyamicin D [29,30,31]. More recent, detailed information about novel bioactive secondary metabolites and their derivatives can be found elsewhere [10,17,31,32].

### 1.3. The Problem of Antibiotic Resistance and the Need to Develop New Compounds That Can Act as Antibiotics

The high rate at which resistant and multi-resistant organisms, or *superbugs*, are emerging is a growing and dangerous problem [33]. An alarming example is MRSA, which causes thousands of infections each year. This is a particular concern in sensitive environments, such as hospitals. The overuse of antibiotics in animals and humans has contributed to this situation [18].

This problem has led to a deeper understanding of resistance systems, also known as the resistome (the set of genes, both inherited and acquired, that confer tolerance to antimicrobials [34]). These systems are developed by microbial communities (such as biofilms) and individual organisms. In their review article on this topic, Penesyan and colleagues distinguished two models to explain these systems ([35] and references therein). **The cellular-level resistance model** implies the existence of mechanisms against different antibiotics. These mechanisms include enzymatic modification of the antibiotic, random mutations, reduced permeability and internalization capacity, active expulsion, and horizontal gene transfer. In the **community-level resistance model**, these authors emphasize the role of organisms such as *P. aeruginosa*. This bacterium protects nearby species in biofilms lacking these compounds thanks to the polysaccharides it secretes. These polysaccharides serve as a protective barrier against aminoglycoside antibiotics, such as tobramycin and gentamicin. Additionally, Penesyan et al. [35] point out that extracellular DNA, which is part of the biofilm matrix [36], may contribute to antibiotic resistance in biofilms. This is likely due to its high negative charge, which could play a role in chelating cationic antibiotics and aminoglycosides. In a more recent study, analyzing changes in the genome of cells dispersed from *Acinetobacter baumanni* biofilms, the same research group showed that, after a 3-day exposure time to subinhibitory concentrations of the antibiotics ciprofloxacin (0.5 MIC) and tetracycline (0.25 MIC), these microorganisms underwent rapid evolution due to the generation of mutations that contributed to antibiotic resistance [37]. Many other publications address the issue of antibiotic resistance, including several extensive reviews [5,38,39].

Correctly understanding these systems is key to overcoming this problem. It also lays the foundation for designing and/or optimizing new and existing strategies. The discovery of new drugs with antimicrobial activity is perhaps more necessary than ever today, yet this is becoming increasingly difficult for several reasons [40,41]. First, natural product discovery is affected by the replication problem of repeatedly discovering the same molecules [42]. This problem is further compounded by the underfunding of these types of projects, which is frequently the case due to their substantial time and financial requirements. Additionally, pharmaceutical companies do not see a clear return on their investments when considering that most projects take about 10 years to develop [43].

The problem of antibiotic resistance has spurred the development of alternative therapeutic approaches beyond traditional antibiotics. These approaches include human monoclonal antibodies, which can exert antimicrobial activity through direct bactericidal action, antivirulence, or toxin neutralization; and antibody-antibiotic conjugates, which facilitate the selective binding of antibiotics to specific antigens and their direct delivery to the site of infection. Other approaches include antimicrobial peptides, which have various mechanisms of antimicrobial action, such as membrane permeabilization and inhibition of cell wall biosynthesis; bacteriophages, which can cause bacterial lysis; and gene therapy, which generates chromosomal alterations that trigger cell death (reviewed in [5]). However, it is important to note that many of them have technical and economic limitations that currently prevent them from being used commercially.

Despite these emerging strategies, it is essential to design and develop new, optimized, and efficient methods for discovering secondary metabolites of microbial origin with pharmaceutical applications due to their antimicrobial activity. The main objective of this work is to describe and analyze the current strategies for achieving this goal. We focus on three strategies: analyzing extreme environments to find new strains or species that produce interesting compounds, using iChip to cultivate previously uncultivable microorganisms, and using metagenomics to circumvent the bottleneck of isolating microorganisms. Additionally, we discuss techniques to avoid replication, such as those based on artificial intelligence, to increase the detection of new compounds that might otherwise go unnoticed.

## 2. Discovery of New Metabolites of Interest from Microorganisms in Extremophilic Environments

One strategy for discovering new metabolites of interest is studying extreme and unexplored habitats. This approach is based on the idea that complex environments favor the development of unique characteristics in organisms that inhabit them. One example is hypersaline marine environments. In 2016 alone, 1277 new marine natural products were described [44].

Many enzymes involved in the production of secondary metabolites of interest or with antibacterial activity are encoded by what are commonly referred to as *biosynthetic gene clusters* (BGCs) [45]. These genomic regions are the ones that are attempted to be localized in the annotation and characterization processes of microorganisms that are candidates to produce bioactive compounds. To detect the product of a given BGC, certain criteria must be met. First, the gene cluster must be transcribed and translated sufficiently. Second, all necessary cofactors and substrates for synthesizing the natural product must be available to the encoded enzymes. Finally, the level of synthesis of the product must be detectable with the available methodology. Finally, an appropriate extraction method for the product must be available. Most BGCs cannot be detected because they do not meet these conditions; thus, they are termed orphan, cryptic, or silent [46]. It should also be noted that the analysis systems used in these searches compare the information with existing databases that have been developed from laboratory results, which correspond to less than 1% of total microbial species [47]. Furthermore, in vitro and in silico analyses often miss some of these clusters because they are silenced under laboratory conditions. This fact often causes replication problems.

**Figure 2:**
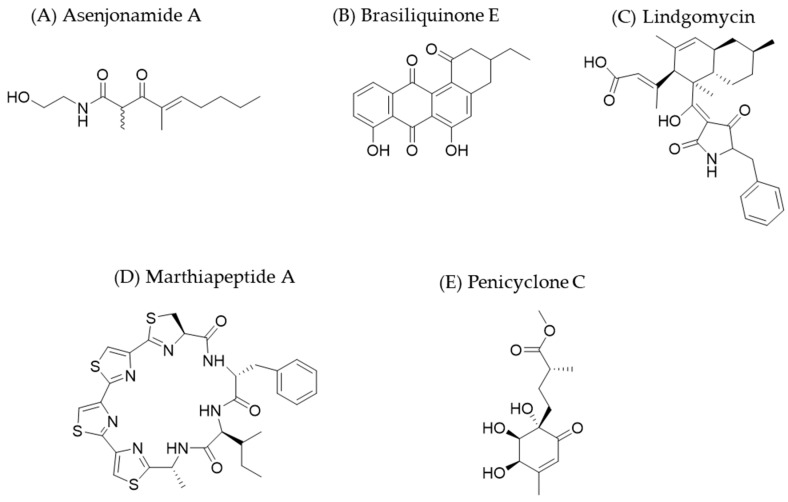
The chemical structures of some new antibiotics discovered from microorganisms in extreme environments.

Abdelkader et al. extracted bacteria from soil in environments where diurnal temperatures of up to 45 °C are reached, such as the Atacama Desert in Chile [48]. Of the strains they tested, one strain of *Streptomyces asenjonii* (KNN 42.f) exhibited antibacterial activity. They carried out antimicrobial susceptibility tests using the agar diffusion method to determine the MIC against a panel of strains (*S. aureus* ATCC 25923, *Bacillus subtilis* NCTC 2116, *Escherichia coli* ATCC 25922, *Enterococcus faecalis* ATCC 10541, and *Mycobacterium smegmatis* ATCC 607). The structure of the bioactive compounds was determined, allowing for the identification of three β-diketones (asenjonamides A–C; see Figure 2). These ketones have been shown to act as metal ion chelators [49], hindering enzymatic activities, such as those of polymerases, that require these ions for their catalytic reactions.

In other studies, using 16S rRNA analysis—a standardized method for identifying microorganisms [50]—Zhang et al. discovered an interesting bacterial strain with anti-tuberculosis activity in one of the climatologically extreme areas of the vast Sinkiang region of China. This strain, *Nocardia* sp. XJ31, was found in soil samples [51]. What was new in their study is the screening strategy they used: the anti-BGC bioassay [52]. This assay uses a *Mycobacterium bovis* strain (BGC Pasteur 1173P2) that constitutively expresses green fluorescent protein (GFP). This assay uses direct fluorescence readings to determine whether there is growth inhibition and thus antibacterial activity. As a result of this experiment, brasiliquinone E (Figure 2) was isolated. This benz[α]anthraquinone may be a promising new alternative for tuberculosis treatment because the main medications for this disease, which are based on isoniazid and rifampicin [53], are becoming less and less effective due to the rise of multidrug-resistant bacteria. Additionally, this finding underscores the significance of diverse microorganisms in producing active compounds against tuberculosis. Approximately 60% of natural products for this disease originate from the genus *Streptomyces* [54].

The search for psychrophilic/psychrotolerant organisms is also interesting. Wu et al. [55] traveled to Antarctic regions to study tetramic acid-derived compounds produced by the *Lindgomycetaceae* fungal family, which was introduced by Hirayama et al. [56]. This molecule is characterized by its pyrrolidin-2,4-dione skeleton. It is a key structural scaffold for many bioactive natural products and exhibits antibacterial, antifungal, antiviral, and cytotoxic properties. From the LF327 strain isolated from this habitat, they obtained lindgomycin (Figure 2). It is a complex derivative of tetramic acid, conjugated with a bicyclic hydrocarbon domain, and exhibits an antibiotic effect against organisms such as *Staphylococcus epidermidis*, *S. aureus*, MRSA, and *Cutibacterium acnes*; however, its effectiveness is lower than that of chloramphenicol.

The research and discovery of new secondary metabolites is also carried out in other largely unexplored environments, such as deep ocean waters. These low-temperature, low-light environments are perfect breeding grounds for organisms that have yet to be identified [57]. One such compound is marthiapeptide A (Figure 2), a cyclic peptide with a tristhiazole–thiazoline structure. It was isolated from the ascomycete *Marinactinospora thermotolerans* SCSIO 00652, which was found at a depth of 3865 m in China. Marthiapeptide A exhibits antibacterial activity against various Gram-positive bacteria and cytotoxicity against several human cancer cell lines [58].

Another example is the discovery of the new metabolites penicyclones A–E (Figure 2), which were isolated from the strain *Penicillium* sp. F23-2 that inhabits depths greater than 5000 m and is effective against *S. aureus* [59]. These compounds are polyketide analogues of ambucic acid, a highly functionalized cyclohexenone [60], which inhibits the quorum sensing of several Gram-positive bacteria [61]. More information about other compounds isolated from deep ocean waters can be found in the work by Tortorella et al. [62].

It is important to note that one difficulty faced in discovering microorganisms in the deep sea is the scarcity of delicate collection methods, as most machinery has been developed by oil companies that generally did not prioritize ecosystem preservation. However, in recent years, prototypes of remote-controlled vehicles capable of extracting samples at these depths have been designed. One example is the Seaeye Falcon of the SAAB company, which has limbs made of soft and delicate materials for better sample collection ([63] and references therein). This technology has been and will be very useful in this type of research.

## 3. Isolation Chip Technique as a Strategy to Cultivate What Seemed Uncultivable

Despite the above findings, a significant challenge remains: the difficulty of growing extremophilic organisms under laboratory conditions. Kaeberlein et al. estimated that 99% of microorganisms cannot be cultured using standard methods [64]. This renders conventional screening techniques impractical [65]. To address this issue, Nichols and his colleagues developed a technique known as the isolation Chip (hereafter iChip) [66].

### 3.1. iChip Description

**Figure 3:**
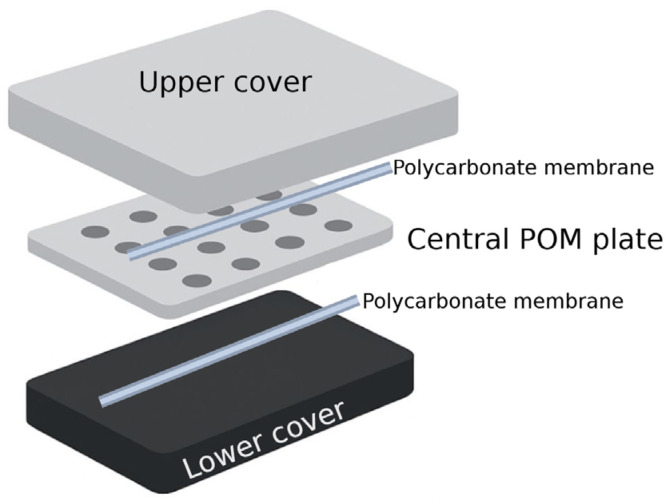
Diagram of the assembled iChip system. It consists of upper and lower covers, semipermeable polycarbonate membranes, and a central polyoxymethylene (POM) plate. Figure was developed in BioRender (https://app.biorender.com/, accessed on 3 July 2025) based on the review by Sherpa et al. [70].

The iChip is an in situ isolation technology consisting of a system composed of plastic plates and membranes. It is designed to isolate environmental microorganisms that can generate substances with antimicrobial properties [67]. The system contains a series of diffusion chambers that allow for the isolation of a single cell per well. It is characterized by a central plate made of hydrophobic polyoxymethylene (POM) (Figure 3). This type of plastic has high dimensional and thermal stability, as well as malleability comparable to metals. This makes it suitable for steam sterilization processes [68]. The plate has two sections with microholes, generally 1 mm in diameter, for a total of 384 holes. Additionally, it consists of impermeable polycarbonate membranes and two plates that fix the membrane and the central plate when screwed together [66].

The working method followed for this system is as follows [66]: First, the soil extract or aquatic region to be analyzed is identified and diluted to a concentration of one cell per hole in the plate. This cell suspension is preferably carried out in a tube containing liquid agar. When the liquid solidifies, the cells are trapped and separated from each other. Polycarbonate membranes are then added to prevent diffusion of the cells into and out of the agar. Finally, the plates are screwed together to seal the system and secure the membrane.

This system prevents the entry of other microorganisms but allows the diffusion of components, such as nutrients. After preparing the iChip, it is placed in the same medium from which the samples were extracted for recovery and growth. Studies by Nichols et al. [66] have shown that this method produces a higher percentage of new organisms than traditional methods, such as Petri dish-based cultures (50% growth recovery versus 1%). A series of conventional cultures must then be performed until the microorganism grows to the desired quantity, a process called domestication [69].

### 3.2. Some Examples of Antimicrobials and Organisms of Interest Discovered Using iChip

One of the most significant discoveries made with this technology was achieved in 2015 by Ling et al. [71]. The researchers analyzed one gram of soil from Maine (USA). After preparing the iChip, they incubated it in the same soil medium, where the sample was extracted for a month. Then, they prepared extracts from the isolates that had grown and screened them for antibacterial activity against *S. aureus*. They identified a new bacterial species in the sample that produced *S. aureus* growth inhibition halos and provisionally named it *Eleftheria terrae*. Sequencing the genetic material and conducting 16S rDNA analysis revealed that this microorganism belongs to a new genus related to *Aquabacteria*. They structurally characterized and named the antibacterial compound produced by this species teixobactin (Figure 1). It is an unusual depsipeptide containing enduracididine, methylphenylalanine, and four D-amino acids. Teixobactin inhibits the synthesis of peptidoglycan. It acts by binding to peptidoglycan precursors, such as lipid II (as occurs with other antibiotics that have lower activity [72]), as well as uncaprenyl-N-acetylmuramic acid-pentapeptide. However, it does not bind to enzymes involved in this process. Teixobactin has been shown to be effective against Gram-positive bacteria, as well as multidrug-resistant strains of *S. aureus* and other difficult-to-treat bacteria, such as *Clostridium difficile*, *Bacillus anthracis,* and *Mycobacterium tuberculosis*.

**Figure 4:**
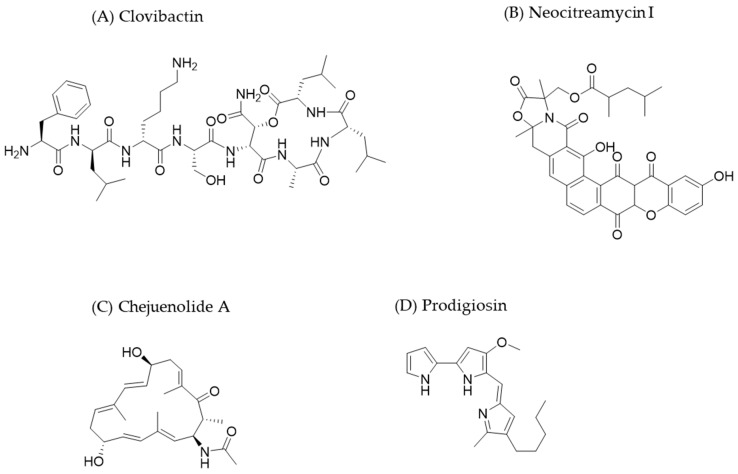
The chemical structures of some new antibiotics discovered through the use of diffusion chambers.

More recently, Shukla et al. [73] discovered another antibiotic using a novel culturing platform derived from the iChip, which was designed by *NovoBiotic Pharmaceuticals* (Cambridge, MA, USA). The compound was obtained from bacteria 99% identical to *E. terrae* found in sandy soil samples from North Carolina and was named *E. terrae* ssp. *carolina*. The antimicrobial compound, clovibactin (Figure 4), is a depsipeptide similar to teixobactin, though it has a shorter linear N terminus where the compound’s two positive charges are located. Clovibactin exhibits antibacterial activity against Gram-positive bacteria, including MRSA and vancomycin-resistant strains. Researchers demonstrated that this antibiotic binds to the pyrophosphate moiety of several cell wall precursors, including lipid II. This region was previously considered an unsuitable target for antibiotics. The aforementioned pharmaceutical company is currently developing it as Novo29.

Previous discoveries made with in situ culture prototypes predating the iChip are noteworthy, such as the diffusion chamber. The diffusion chamber has the same rationale as the iChip, but it cannot isolate single cells. One notable discovery was an unknown strain of the genus *Nocardia* that produces the compounds neocitramycin I and II (Figure 4), polycyclic xanthones active against Gram-positive bacteria ([70] and citations therein).

Despite being cultivable, the iChip is effective in characterizing bacterial species that can be better studied through the recovery and subsequent analysis it provides. He et al. [74] used this tool to discover the *Hahella chejuensis* NBU794 strain in mangrove soil from the Dongzhai Port (Haikou, China). They identified two groups of new metabolites from this strain: chejuenolide A–C (Figure 4) and nine prodiginine derivatives (including the lead compound prodigiosin (Figure 4) and two new compounds, 2-methyl-3-pentyl-4-O-methyl-prodiginine and 2-methyl-3-octyl-prodiginine). Prodiginines are tripyrrole compounds with immunosuppressive and anticancer activity [75]. He and colleagues [74] demonstrated that prodigiosin and 2-methyl-3-heptyl-prodiginine were the most active compounds against *Escherichia coli*, *Salmonella paratyphi B*, *S. aureus*, *Bacillus subtilis*, and *Candida albicans.* Despite the limitations of this study due to a lack of genomic data and large-scale fermentation techniques, it represents a step forward for future studies and may contribute to combating *superbugs*.

Interestingly, this technique is not only of interest to the pharmaceutical industry; it has also been used to cultivate organisms with bioremediation capacity [76].

### 3.3. iChip Modifications for Improved Performance

**Figure 5:**
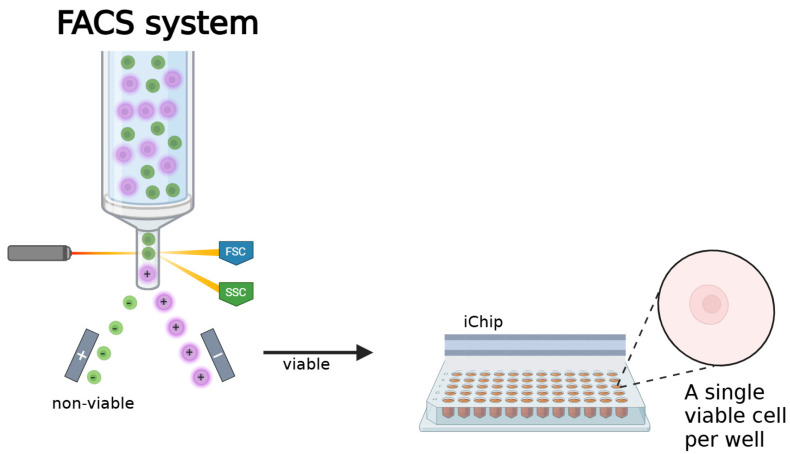
FACS-iChip system, in which, before culturing the sample on the iChip, live cells are separated from dead cells using flow cytometry. Figure was created using BioRender (https://app.biorender.com/, accessed on 3 July 2025) with the information provided by Liu et al. [78].

The iChip system is still being developed and improved to substantially increase its performance. One issue with the iChip is that it is unclear whether the correct distribution has been achieved. Diffusion chambers may contain zero, one, or several cells, some of which may be nonviable [77]. One of the most interesting modifications in recent years to solve this problem is the addition of a fluorescence-activated cell sorter (FACS) (Figure 5). This flow cytometry system [78] uses a laser to preselect live and dead cells, ensuring that only one viable cell is present in each well.

Specific adaptations have also been made to the iChip to accommodate different conditions. To cultivate species that were initially unable to be cultured from hot spring samples in Tengchong (China), Zhao et al. [79] slightly altered the size and shape of the chip. They also replaced the usual solidifying agent, agar, with gellan gum. This was necessary because agar melts when attempting to cultivate thermotolerant microorganisms at very high temperatures. The screw-on plates were also eliminated, and the membranes were fixed to the POM plate with glue. This change was intended to increase the surface area of contact and facilitate the diffusion of nutrients and growth factors in complicated environments such as hot springs. The results of this research were positive, raising hope for the future use of this technique in analyzing extreme environments.

### 3.4. Use of Siderophores as Growth Enhancers

Some researchers have observed that certain microorganisms only grow in the presence of other species living in the same environment that secrete growth factors recognized by those species [80]. D’Onofrio et al. [81] analyzed these helper strains in some isolates from marine sediment biofilms and demonstrated that the growth factors they produced were novel acyl-desferrioxamine siderophores [82]. According to these results, some bacteria do not need to produce their own siderophores because they can use those produced by other species. The absence of these growth factors in some non-culturable bacteria may be due to evolutionary loss or distinct regulation, as 16S rRNA analysis revealed a high degree of similarity between these bacteria and other culturable strains in their environment. In practice, this means the growth of these bacteria can be stimulated by adding external siderophores or by introducing artificially high concentrations of iron. This opens up the possibility of searching for new compounds by combining this process with in situ culture techniques, such as iChip. However, scaling up these organisms for mass production on an industrial scale presents many challenges.

## 4. Metagenomics to Analyze Complex Microbial Communities and Discover Natural Products of Interest

A frequently used strategy to avoid problems when isolating unknown organisms (and also as a preliminary step to iChip) is to analyze the entire genome of a complex environmental or clinical sample [83]. This is the objective of metagenomics, which Sleator et al. [84] defined as the functional and sequence-based analysis of the collective microbial genomes (or microbiome) in a specific environment or environmental niche.

### 4.1. Working Methods in Metagenomics

Currently, there are two main strategies used in metagenomics: untargeted and targeted analysis [85,86]. In untargeted (shotgun) metagenomics, all of the DNA present in an environmental sample is sequenced randomly and extensively. This approach generates millions of sequence fragments that are assembled and analyzed using advanced bioinformatics tools. The resulting data allow for the taxonomic and functional characterization of the microbiome present in the sample. They also enable the identification of individual genes, complete metabolic pathways, and biosynthetic gene clusters. This is especially relevant for natural product discovery. However, this approach relies on in silico predictions and does not guarantee that the identified genes are active or can be expressed; therefore, the findings must be validated through additional techniques. In contrast, targeted metagenomics analyzes specific genes or regions of the genome. The best-known methodology is amplicon sequencing, which begins with PCR reactions. Primers are used to amplify specific genes, such as antibiotic resistance genes or the gene that encodes 16S rRNA. Analysis of the latter is generally the first step, as it serves as a phylogenetic marker in a given sample [87]. This allows researchers to proceed with the search for new natural products.

Metagenomics is essential for identifying and characterizing microorganisms in samples. It has substantially improved with the implementation of next-generation sequencing (NGS) techniques, which enable massive, high-precision sequencing [88,89]. Second-generation sequencing technologies, such as Illumina’s sequencing-by-synthesis method using reversible dye terminators and Thermo Fisher’s (Waltham, MA, USA) Ion Torrent method, which detects hydrogen ion release during DNA synthesis, are characterized by their high capacity and low cost. These technologies rely on massive parallel amplification to sequence short DNA fragments. These short reads, ranging from 100 to 300 base pairs, are useful for identifying conserved genomic regions, making them ideal for amplicon sequencing [83]. Third-generation sequencing platforms, such as those developed by Pacific Biosciences (PacBio) (Menlo Park, CA, USA) and Oxford Nanopore Technologies (Oxford, UK), allow for long reads ranging from a few to tens of kilobases [90]. These platforms are essential for expanding our ability to detect the dynamics of antimicrobial resistance and provide early warning signals for potential future threats. Third-generation sequencing platforms also play a crucial role in annotating new genes, such as those belonging to BGCs.

Ranjan et al. [85] conducted a comparative study of two strategies for analyzing the human fecal microbiota. According to the authors, 16S amplicon analysis is the most widely used method in this type of research and has several advantages. These advantages include low-cost bioinformatics analyses that can be performed using established tools and workflows, which facilitate implementation in laboratories. Additionally, a large amount of reference data is available in public databases. However, the researchers’ results revealed that whole-genome shotgun analysis allows for the identification of a greater number of species per read (nearly double). This approach enhances the detection of bacteria and organisms belonging to other kingdoms, such as viruses and fungi. It also enables the direct identification of genes present in the microbiome. This overcomes the limitation of the 16S method, which only offers taxonomic predictions based on conserved regions of ribosomal DNA. Finally, the longer contigs provided by the shotgun approach increased the accuracy of species detection compared to matching and assembling shorter fragments.

### 4.2. Examples of Metagenomics Applied to the Discovery of Natural Products

**Figure 6:**
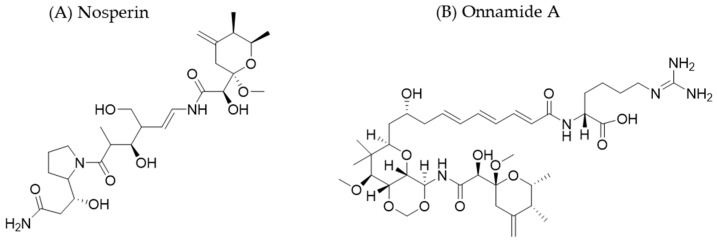
The chemical structures of some bioactive natural products discovered using metagenomic approaches.

In lichens, integrated symbioses occur between the heterotrophic filamentous fungi (mycobionts) and cyanobacteria or coccoid-shaped green algae (photobionts). Kampa et al. [91] analyzed symbiotic communities attached to the lichen *Peltigera membranacea*. Investigating these communities presents a fundamental challenge. Photobionts can typically be grown in pure cultures, but most mycobionts (almost all of which belong to the *Ascomycota* phylum) cannot be propagated in vitro using conventional methods. Furthermore, intact lichens cannot be maintained artificially for long periods. To circumvent these issues, the researchers used metagenomics approaches. Specifically, they sequenced the entire genome of the *P. membranacea* lichen and detected candidate clusters of genes encoding polyketide synthases in the photobiont *Nostoc* sp. One of these clusters had homology with those involved in the biosynthesis of pederin-type family compounds. Pederin family members possess antitumor activity due to their ability to inhibit eukaryotic ribosomes [92]. Further analysis of this organism enabled the researchers to identify nosperin (Figure 6). This is a new polyketide belonging to this family that is composed of pederin and a proline-containing terminal moiety [91]. The latter region was not found in other products of this group. The results of this study reveal the potential of exploring other symbioses for drug discovery.

The marine sponge *Theonella swinhoei* is an emblematic example of the biotechnological potential of the marine microbiome. It harbors a diverse consortium of microorganisms, many of which cannot be cultivated in a laboratory setting [93]. In 2014, Wilson and his colleagues published a study aimed at identifying the microorganisms responsible for producing bioactive compounds previously isolated from the consortium of species associated with *T. swinhoei* in Hachijō-jima (Japan) [94]. The researchers took two parallel approaches. First, they used a single-cell approach. After separating the bacteria by density using differential centrifugation, they observed fractions enriched with 2–3 µm filamentous bacteria that fluoresced under UV light under a microscope. These bacteria were morphologically similar to *Candidatus Entotheonella palauensis*, a symbiont previously found in *T. swinhoei* in the Palaos archipelago. The bacteria were sorted individually into multiwell plates using fluorescence-activated cell sorting. Then, the researchers amplified their DNA using primers specific for the 16S rRNA gene and genes involved in biosynthetic pathways. Positive signals were obtained in 46–71% of the wells, depending on the primers used. These results provided strong evidence linking *Entotheonella* sp. to the production of onnamides (Figure 6) and polytheonamides, the two main classes of bioactive compounds present in the sponge. The second approach, which was based on metagenomics, sought to validate these findings through multiple rounds of sequencing using different second- and third-generation platforms. Subsequent bioinformatics and functional analyses identified a single microbiome member, *Entotheonella factor* TSY1, as the source of virtually all the bioactive polyketides and peptides associated with *T. swinhoei*. This discovery highlights the importance of *Entotheonella* in marine secondary metabolism and emphasizes the value of combining single-cell and metagenomic approaches to study non-culturable microbial diversity and its pharmacological potential.

### 4.3. Problems Associated with Metagenomics

Metagenomics is a multipurpose technique, but it can present problems related to the tools required for DNA analysis of samples, such as costly sequencing machinery, specialized labor, and logistical difficulties. There are also important limitations. These include low resolution, biased classification of short sequences, and functional errors due to the misinterpretation of genes. Other shortcomings include the difficulty of obtaining high-quality DNA from extremophiles due to their cellular resistance to cleavage and possible gene cloning errors in hosts such as *E. coli* or other biological systems, where bioactive compounds are not always produced correctly [95]. Additionally, the presence of environmental extracellular DNA in samples can complicate metagenomic analyses and lead to an overestimation of diversity. In their review article, Bairoliya et al. describe the importance of considering this factor when interpreting experimental results and outline methods for extracting and quantifying this DNA [96]. A significant drawback is the quantity and quality of the data analyzed using bioinformatics tools after sequencing is complete, as well as the limited information about antibiotic resistance genes in existing databases [83]. During metagenomic analysis development, false positives and false negatives may be encountered. False positives are identified based on experimental factors, such as sample contamination and DNA manipulation during extraction, handling, preservation, and sequencing. Computational factors related to reference-based metagenomic profilers also contribute to false positives [97]. These types of problems can also result in false negatives.

## 5. Artificial Intelligence as a New Strategy to Avoid Replication and Accelerate the Search for New Compounds of Interest

**Figure 7:**
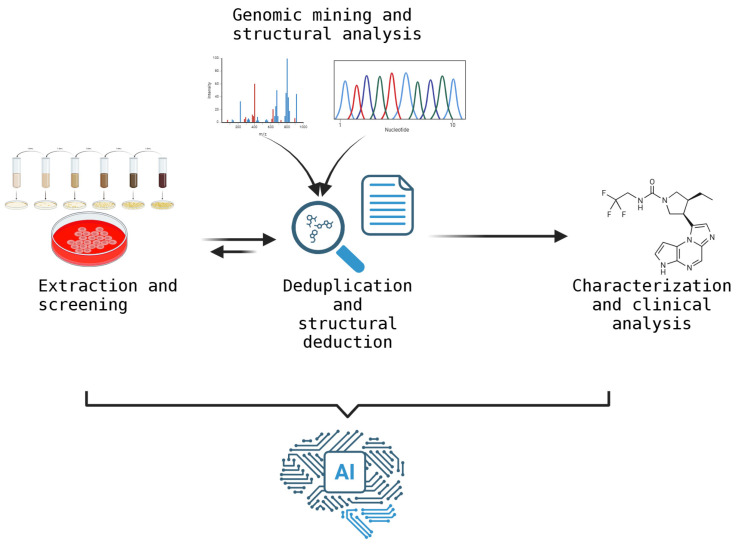
Summary diagram of the process for searching for new natural products. The diagram illustrates how artificial intelligence can optimize each level and step of the process. This figure was created using BioRender (https://app.biorender.com/, accessed on 3 July 2025).

Until now, all the discussed strategies and discoveries have been based on the first step of the research process: extracting and cultivating the sample of interest. However, a significant problem has emerged, causing numerous setbacks for researchers. This problem is called replication [98], which is the continuous discovery of the same compounds over and over again. To address this issue, techniques known as deduplication have been developed and implemented [99,100]. These strategies quickly identify previously described molecules, avoiding redundancies and streamlining the process. In recent years, developing these strategies has required computational improvements and the use of artificial intelligence (AI) [101,102] (Figure 7).

AI uses machines, primarily computer systems, to mimic human cognitive processes, such as learning, reasoning, perception, and language comprehension. Gangwall and Lavecchia [102] published an extensive review describing how AI can help researchers identify natural products. The rise of chatbots like ChatGPT (https://chatgpt.com/) has given rise to new AI applications, including computational models and machine learning (ML). The primary objective of ML is to empower computers to learn from data and generate predictions. These tools are useful for creating predictive models that establish relationships between molecular structure and biological activity. Additionally, ongoing work involves integrating AI with mass spectrometry, chromatography, and nuclear magnetic resonance analyses. This allows AI to quickly identify and analyze samples and generate data for researchers. Deep learning (DL), a subfield of machine learning, is designed to understand complex data representations and is useful for molecular design. Computer vision is an essential AI component for analyzing visual data from various natural sources. Integrating it with spectroscopic methods helps characterize bioactive compounds.

Drug discovery based on secondary metabolites presents various organizational and accessibility challenges. For example, databases such as PubChem often lack information about natural products. Additionally, intellectual property issues render many tools and databases inaccessible to the average researcher [98]. To address these issues, the MELODDY project was recently developed [103]. The project uses federated learning, an artificial intelligence technique that enables machine learning models to be trained using data distributed across different locations without centralizing it. This allows third-party data to be shared without compromising its sensitivity, thus avoiding intellectual property issues.

**Figure 8:**
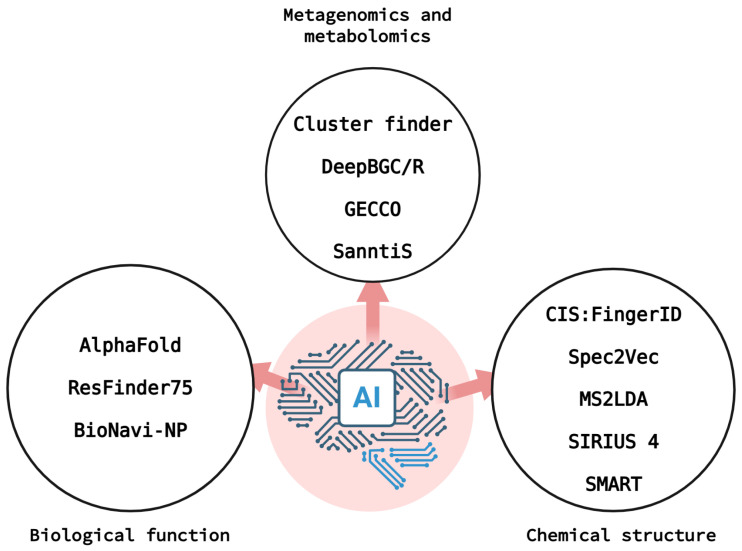
Artificial intelligence applications in the discovery of novel natural products. These include (1) metagenomics and metabolomics; (2) biological function prediction; (3) chemical structure elucidation. The figure was created in BioRender (https://app.biorender.com/, accessed on 3 July 2025).

Below, we discuss AI-based strategies that may be useful for identifying natural products of interest to the pharmaceutical industry. These strategies can be applied at various levels (Figure 8).

### 5.1. Artificial Intelligence in Genomic Mining and Metabolomics

As previously mentioned, analyzing the sequence of obtained samples for known biosynthetic gene clusters is often necessary to deduce the possible bioactive characteristics of their metabolites. To this end, *antiSMASH* and *PRISM* are commonly employed. *antiSMASH* detects and classifies BGCs in genomes [104], and *PRISM* predicts the chemical structure of secondary metabolites based on the genome [105]. These bioinformatics tools are called rule-based methods because they follow logical rules imposed by humans and cannot learn independently. While this makes them extremely useful for detecting known BGCs, they are not flexible enough to predict new ones [102,106]. To address this issue, AI-based strategies are now available. The best-known tools of this type are *ClusterFinder*, which uses machine learning to detect unknown BGCs based on genetic patterns [107]; *DeepBGC*, which uses DL technology to detect and classify BGCs more accurately [108]; *eDeepBGS,* which reduces high positive rates and increases sensitivity in identifying these clusters compared to *DeepBCG* [109]; *GECCO*, which searches for larger, more complex structures called superclusters or composite clusters [110]; and *SanntiS*, which uses structural deep learning to predict the exact type of metabolite produced by a set of genes [111].

**Figure 9:**
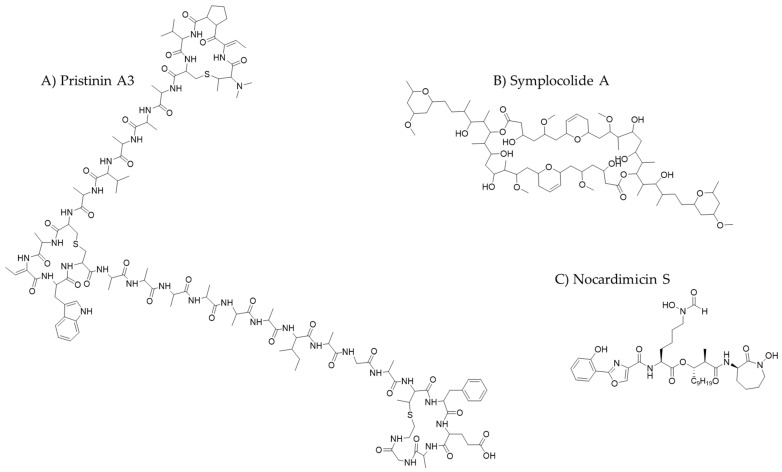
The chemical structures of some new antibiotics discovered with the aid of artificial intelligence.

Novel ribosomally synthesized and post-translationally modified peptides (*RiPPs*) are a large, continuously growing superfamily of natural peptides. Many RiPPs have not yet been biochemically characterized, and most of their discovery has been in silico. This superfamily includes redox cofactors, chalkophores, and siderophores, among others. *RiPPs* exhibit diverse bioactivities, including antifungal, antiviral, antiparasitic, antitumor, and analgesic therapeutic effects [112,113]. New AI-based tools can be used to detect RiPP biosynthetic gene clusters. One such tool is *DeepRiPP*, which uses ML to integrate genomics and metabolomics to aid in the identification and isolation of RiPPs [114]. Another approach is *decRiPPter* (*Data-driven Exploratory Class-independent RiPP TrackER*) [98,115]. Using this platform, researchers identified 42 new *RiPP* candidate families from 1295 *Streptomyces* genomes. The researchers also studied one of these families in depth and discovered a representative of a new group of therapeutically active lanthipeptides, pristinin A3 (Figure 9). While these resources often generate numerous false positives and negatives, they provide greater flexibility and the capacity to predict unknown clusters. These tools are expected to provide a wealth of information in the near future [98].

### 5.2. The Use of Artificial Intelligence for Predicting Chemical Structures

AI can be used to predict metabolites from chromatographic and spectrometric profiles obtained by mass spectrometry or nuclear magnetic resonance. Despite the lack of development due to insufficient annotated spectral data and generalized criteria for published data formats [116], AI is being used for tasks such as peak integration in chromatograms, retention time prediction, and processing missing data [117,118].

As the volume of mass spectrometry data has increased, several metabolomics platforms have emerged. These platforms use predictive models and automated analysis on large amounts of metabolic data. One example is *MetaboAnalyst6.0* software [119]. Other ML-based platforms include *CSI:FingerID* [120], *MS2LDA* [121], *SIRIUS 4* [122], and *Spec2Vec* [123]. These tools connect tandem mass spectrometry spectra with specialized molecular databases to identify the structures of natural products [101]. However, the quality of the spectrum and database affects interpretation, which limits their use with new compounds.

Finally, artificial intelligence has been used in nuclear magnetic resonance (NMR) spectroscopy to improve structural prediction and annotation based on data from this technique. The *SMART* (Small Molecule Accurate Recognition Technology) prototype was developed using DL. It learns how to cluster similar compounds by being trained on a database of spectra of 2054 compound spectra [124]. *CASE* (Computer-Assisted Structure Elucidation)-type programs generate a probability-ordered list of all possible structures from an NMR dataset, thereby reducing errors in structure proposals [125]. The authors describe four different CASE programs that provide planar skeletal structures: *ACD labs structure elucidator*, *Bruker CMC-se program*, *Mestrelab MNova structure elucidation program,* and *Nuzillard’s LSD program*. A new CASE-3D strategy that uses isotropic and/or anisotropic NMR has also been proposed to deduce the preferred conformation [126]. This strategy forms the basis of the MNova Stereofitter program, which computes the probability of 3D structural configurations and/or conformations.

These programs have enabled the discovery of new classes of natural products, such as symplocolide A (Figure 9). This compound was found in a crude extract of the marine cyanobacterium of the genus *Symploca* using *SMART 2.0*, an improved version of the *SMART* prototype that allows the rapid prioritization of structurally novel and interesting natural products [127]. Symplocolide A is a chimeric macrolide similar to swinholide, which is a compound isolated from marine sponges that inhibits actin polymerization.

### 5.3. Artificial Intelligence for Determining Biological Functions

In many cases, the raw samples extracted from a natural product are insufficient to elucidate its biological function or to provide data to train ML models. Currently, bioactivity assays based on the 3D structure of a compound predicted by AI models using only its sequence are available. Thus, tools such as *AlphaFold* [128] allow the three-dimensional structure of a protein of interest to be deduced from its amino acid sequence [129]. This advance enables molecular docking, a computational technique that simulates how a small molecule can bind to a target protein, to determine its potential therapeutic function when this is unclear or unknown [130]. However, docking has limitations. It only predicts potential affinity, not actual activity, and has a high false-positive rate.

On the other hand, AI is being trained using databases associated with antibiotic resistance genes. These databases include the *National Database of Antibiotic-Resistant Organisms* (*NDARO*, created in 2019 by the *National Center for Biotechnology Information*, *NCBI*); the *Comprehensive Antibiotic Resistance Database* (*CARD*, [131]); and *ResFinder75* [132]. These strategies are based on the idea that if a bacterium has certain resistance genes, the AI can infer that it produces an interesting antibiotic or compound. Then, the AI can deduce the conditions under which these genes are activated and determine when and where the substance is produced. Advances are also being made in analyzing and predicting the biosynthetic pathways of natural products, many of which were previously unknown. This allows us to correctly understand and analyze new proposals for secondary metabolites. To this end, Zheng and his colleagues developed *BioNavi-NP*, an innovative DL-based tool designed to predict the biosynthetic pathways of natural products and structurally related compounds [133].

Ultimately, these tools all contribute to the goal of facilitating and significantly improving the elucidation and reconstruction of complex metabolic pathways. This allows for a deeper understanding of natural product biosynthesis and opens new avenues for drug discovery. It is also worth mentioning the relevance of using some of these strategies to successfully identify and characterize a natural product of interest. This was recently demonstrated with nocardimicins S-W (Figure 9). A combined strategy of 2D NMR, metabolomics, and genome mining was used to discover these five new nocobactin-type lipopeptides with cytotoxic activity in psychrophilic *Nocardia* sp. L1016 [134].

### 5.4. Current Challenges and Problems Related to Machine Learning

Gangwall and Lavecchia [102] pointed out that the main limitations of machine learning stem from the following factors: limited data availability, the complexity of natural products, hurdles in synthesis, biological complexity, and interpretability and transparency issues. These factors all contribute to reducing the reliability of AI predictions. Below, we will analyze some of these aspects in more detail.

One of the main challenges these tools face is the presence of insufficient and unusable data. This is due to a lack of content in available databases, particularly of mass spectrometry and nuclear magnetic resonance spectra. Other challenges include inadequate references and legal barriers to data sharing, such as intellectual property issues. Additionally, a wide variety of information is present in different formats. A few years ago [135], the need to standardize these data for AI to correctly read and learn from them was pointed out, although this issue remains unsolved.

Although artificial intelligence has improved drug discovery, it still faces significant challenges in the synthesis of natural products due to their structural complexity. Thus, improved algorithms, the integration of diverse data, and new technologies, such as quantum computing and advanced generative models, are necessary to overcome the limited diversity and challenging synthesis of proposed molecules. However, this requires very powerful and expensive hardware, which poses a challenge to the implementation of AI in many research laboratories. Nevertheless, accessible tools exist, such as *AiZynthFinder* [136]. This flexible and robust tool is useful for planning synthesis routes and designing strategies to build complex molecules from simpler compounds. Later versions of this open-source software have been released. AiZynthFinder 4.0 [137] includes policies for filter reactions, support for one-step retrosynthesis models, a scoring framework, and additional search algorithms. These improvements reduce the limitations of the first version.

Finally, the intricate network of molecular interactions in biological systems, known as biological complexity, presents an obstacle to the ability of AI to predict the efficacy and safety of newly identified compounds [102].

## 6. Conclusions and Future Perspectives

**Figure 10:**
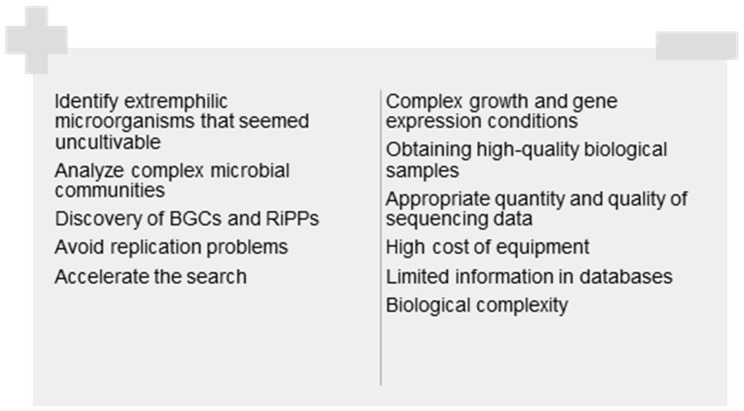
Advantages and constraints of the strategies described in this revision for the discovery of new microbial metabolites with antibiotic activity.

In the current biomedical landscape, the discovery of new microbial metabolites is crucial yet challenging. These compounds have diverse applications; however, this study only considers those that may have potential as antimicrobial agents in light of the growing problem of multidrug-resistant microorganisms. Emerging techniques such as iChip technology, the analysis of extreme environments, metagenomics, and artificial intelligence open up new possibilities and have been shown to circumvent major challenges to some extent. However, the high replication of known compounds, insufficient funding, and technical limitations in sample collection and analysis persist (Figure 10). Information gathered during the development of this study allows us to conclude that a multidisciplinary approach combining technological innovation and collaboration between biotechnologists, bioinformaticians, chemists, and physicians is required in the future. Strategies based solely on new microbial metabolites are not the only way to identify new antimicrobial agents. Other alternatives, which are the subject of intense research, include strategies based on antibodies, antimicrobial peptides, bacteriophages, and gene therapy. A review of these options can be found in the work by Ho et al. [5]. It is also crucial to implement policies that support research. One relevant problem is the lack of investment in development.

## Data Availability

No new data were created or analyzed in this study.

## Abbreviations

The following abbreviations are used in this manuscript:

AI	Artificial Intelligence.
BGC	Biosynthetic Gene Cluster.
DL	Deep Learning.
FDA	Food and Drug Administration.
MIC	Minimal Inhibitory Concentration.
ML	Machine Learning.
MRSA	Methicillin-resistant Staphylococcus aureus.
NMR	Nuclear Magnetic Resonance.
RiPPs	Ribosomally Synthesized and Post-translationally Modified Peptides.
